# Challenges and best practices for moving forward in interprofessional collaboration in critical care units: nurses’ perspectives

**DOI:** 10.1186/s12912-025-02860-0

**Published:** 2025-03-25

**Authors:** Amina Hemida Salem Ghattas, Hala Ahmed Abdou

**Affiliations:** 1 Critical Care & Emergency Nursing Department, Faculty of Nursing, Alexandria University, Alexandria, Egypt; 2 Nursing Administration Department, Faculty of Nursing, Alexandria University, Alexandria, Egypt

**Keywords:** Interprofessional collaboration, Critical care units, Challenges, Best practices

## Abstract

**Background:**

The positive outcomes for critically ill patients rely on effective interprofessional collaboration (IPC), which depends on the collective expertise and skill of the critical care team (CCT) working cohesively and collaboratively to provide patients with high-quality, safe care.

**Aims:**

(1) To determine the challenges to interprofessional collaboration in critical care units as perceived by the diploma versus. baccalaureate nurses (2) To explore best practices suggested by nurses for improving IPC.

**Methods:**

A descriptive design using a four-point scale survey and two open-ended questions was employed to collect the data from 125 nurses in three critical care medicine units at a university hospital. Quantitative data were analyzed using t-tests, one-way ANOVA, and Pearson correlations, with 𝑝 < 0.05 considered statistically significant. Open-ended questions were analyzed by content analysis.

**Results:**

The results demonstrated a substantial difference between nurses who graduated from nursing faculties and hold baccalaureate degrees and nurses who graduated from nursing institutions and hold diploma degrees in agreement and satisfaction with the relations and collaboration with the physicians. Additionally, a significant correlation was seen between the level of nurses’ education and the limited time caused by heavy workloads and the nurses’ perspectives toward their relations with physicians. A content analysis of the nurses’ texts revealed that five categories have emerged as nurses’ suggestions to keep up the best practices for moving forward in the nurse-physician collaboration: changing the physician’s attitude toward the nurses, improving communication skills, role clarification, interprofessional conflict resolution, and support and motivation.

**Conclusions:**

On the one hand, nurses with baccalaureate degrees expressed lower satisfaction with IPC. On the other hand, five categories were suggested by the nurses as best practices for moving forward in their collaboration with the physicians, these include physician attitudes toward the nurses, communication tools and skills, nurses’ roles clarification, interprofessional conflict resolution, and support and motivation. Implementation of the results of the current study will improve patient outcomes by preventing oversights, medication errors, and redundant interventions. In addition to creating relaxed working conditions, improving the morale of the healthcare team, job satisfaction, and decreased staff turnover.

**Supplementary Information:**

The online version contains supplementary material available at 10.1186/s12912-025-02860-0.

## Introduction

Interprofessional collaboration among critical care professionals is essential for providing safe and effective care for critically ill patients who are vulnerable to death at any time due to their life-threatening conditions. Interprofessional collaboration, in combination with high levels of assertiveness and cooperation, is a process whereby professional groups work positively together, bringing together the valuable and unique contributions of experts in the field of patient care and achieving common objectives through joint communication and joint decision-making. Moreover, a true IPC is relational and requires skills in communication, trust, knowledge, shared responsibility, mutual respect, optimism, and collaboration [1–9].

There is an important gap in the literature on nurse/physician collaboration. Nurses’ perception of collaboration differs depending on the clinical practice area. Critical care nurses reported greater satisfaction with nurse/physician communication than nurses working in general care areas, however, disparities remain concerning the perception of communication [10]. When nurses and physicians are surveyed about their perception of collaboration, physicians consistently report a higher level of collaboration than nurses [2, 4, 10, 11]. In addition to creating a healthy work environment, units with effective nurse-physician collaboration benefit patients Units with superior nurse-physician collaboration were significantly associated with lower rates of hospital-acquired pressure injuries and fewer patient falls [12].

Effective communication is a critical component of collaboration. In ICUs where nurses and physicians engage in effective collaboration, rates of central line-associated bloodstream infections and lower rates of ventilator-associated pneumonia are lower. These studies highlight the importance of nurse-physician collaboration as it relates to patient safety and improving patient outcomes. However, lack of teamwork, communication, and collaboration is a major contributor to medical errors, unsafe care, and poor patient outcomes. Moreover, perceptions of hostile working conditions, low morale, job dissatisfaction among healthcare professionals, increased staff turnover and moral distress have been associated with ineffective cooperation. To this end, new mandates are emerging to focus attention on strategies to develop IPC as a component of safe patient care within healthcare organizations [5, 9, 13, 14].

Critical care teams (CCTs) can engage in four professional interactions within a critical care unit: networking, teamwork, collaboration, and coordination. These interactions depend on the degree of shared vision among team members, context, medical problem to be addressed, and urgency for resolution. A successful team relies on strong communication and unified action, such as the cooperation needed in a cardiac arrest code team. A well-functioning clinical trial involves all team members feeling valued and empowered to contribute; clinical trials are purposefully created through the collaboration of doctors and nurses, establishing an environment where all team members feel comfortable sharing their insights and expertise in patient care. When there is a lack of psychological safety for collaborative problem-solving, there is a greater chance of overlooking important information, leading to potential errors in decision-making [10, 15, 16].

Due to the dual nature of their roles, critical care nurses operate at a crossroads between teams, and organizational and management factors. Working at the central point of patient care, critical care nurses collaborate closely with the interprofessional CCT to deliver continual monitoring, problem-solving, decision-making, and advocacy for their patients around the clock. The nurses are subject to organizational rules as employees of the hospital and direct beneficiaries of managerial decisions relating to nurse staffing ratios. Critical care nurses may have to balance the interests of their colleagues in critical care, especially physicians and patients, families, and administrators, regularly. They have experience working interprofessional, given their dual role as nurses in critical care [16–20].

The importance of collaboration, communication, and cooperation in the provision of medical care is often emphasized by physicians and nurses. However, how all professions define these essential components of a practice environment has not yet been shown to be established. The two core competencies needed for patient-centered cooperation are to understand and appreciate the roles and responsibilities of health professionals, as well as to communicate efficiently. Studies in which doctors and nurses were asked about collaboration and communication within their units suggest that the views of physicians and nurses tend to be very different. The complexity of the delivery of critical care services requires continuous integration of skills and knowledge from a range of professions [7, 21].

Improving collaboration facilitation and eliminating barriers to collaboration could enhance clinical practice. Achieving this goal may involve enforcing regulations that require interprofessional healthcare education throughout undergraduate and postgraduate studies. This approach would help shift the mindset of working in isolation by training future professionals to collaborate while considering gender, age, and educational background. Several areas, including the perception of health professionals, are highlighted in emerging research, highlighting the challenges and opportunities to improve interprofessional collaboration between Community colleges and universities [7, 21–23]. This study aims to determine IPC’s challenges and explore the best practices for improving it as perceived by critical care nurses.

### Significance of the study

Nurses and physicians constitute the two main groups of healthcare providers providing direct inpatient care. Many studies in the critical care units have found that effective collaboration between nurses and physicians is crucial for ensuring patient safety and it is one of the essential indicators of the quality of patient care and, enhances workplace quality, cost-effectiveness, and health professionals’ level of satisfaction [4, 24–27]. Despite these positive effects of IPC, nurse-physician collaboration remains a big challenge. Identifying the challenges nurses and physicians face when collaborating can help develop strategies to address and minimize these challenges. Addressing these challenges will have a positive impact on the collaboration between nurses and physicians, ultimately leading to better patient outcomes. Additionally, this study will help to identify the strategies that could enhance or improve the Interprofessional Collaboration between physicians and nurses while interacting with each other regarding patient care during their professional obligations.

### Operational definitions

The study includes two categories of nurses, the following is an operational definition that differentiates between these two categories:

### Diploma nurses (nurse technician)

The nurses who have hold diplomas have graduated from the Nursing Institute (which is not considered a higher education). In terms of scientific background, in short, we find that the focus of the nursing diploma is on aspects of applied and technical skills required to carry out specific tasks during clinical practice in nursing, Therefore, the role of the nursing technician is to provide direct nursing care. In the Nursing Institute, the study period is only two years and 6 months as an internship. The graduate is called a nursing technician.

### Baccalaureate nurses (nurse specialist)

The nurses who have hold baccalaureate degrees have graduated from the Colleges of Nursing (higher education). In terms of scientific background, their scientific knowledge is more profound and comprehensive to enable them to practice nursing care with professional independence. Through applying the concepts, stages, and elements of the nursing process, and providing comprehensive and specialized nursing care. Contributing to administrative supervision, continuing education and training programs, and improving the quality of Health services, as well as participating in scientific research and applying its results. Additionally, the number of years of study in a College of Nursing is 4 years, and one academic year is an internship. The graduate is called a nursing specialist.

#### Aims

1. To determine the challenges to interprofessional collaboration in critical care units as perceived by the diploma versus. baccalaureate nurses.
2. To explore best practices suggested by nurses for improving IPC.

#### Research questions

- What do nurses perceive as challenges to effective nurse-physician collaboration in the Critical Care Units?
- What best practices do nurses suggest that could enhance collaboration between nurses and physicians in the Critical Care Units?

##### Design

A descriptive research design using a four-point Likert scale was used to collect the quantitative part of the study. In addition, two open-ended questions were used to provide a better and deeper understanding of the nurses’ perspectives by allowing nurses to express themselves freely, allowing researchers to gain insights into the underlying reasons behind their perceptions and suggestions processes.

##### Settings

This study was conducted in three critical care medicine units at Alexandria Main University Hospital (AMUH) namely: the general ICU Unit I which includes 8 beds; Unit II 12 beds, Unit III 18 beds. These units receive patients with various disorders in the acute stage of illness. The patients are admitted either directly from the emergency department, or transferred from other departments of the hospital, or other hospitals.

##### Sample

Nurses who worked in the three selected units were invited to participate in the current study. Nurses were considered eligible if they were voluntarily willing to participate in the study, had six months or more working experience in the selected settings to be able to better assess this relationship. and provided direct patient care. Head nurses, nursing managers, and internship nurses were excluded. Internship nurses were excluded because they were considered trainees and did not have a direct relationship with the physicians. The sample size was calculated by using an online equation to determine a representative sample for both nurses and physicians who are working in critical care units with a confidence level of (95%) and a desired confidence interval of (5%). Based on the calculated equation, the recommended sample size of this study was 116 nurses. The sample size was increased to 125 given the possible number of nurses leaving.

### Tool: “interprofessional collaboration: key challenges and best practices”

This tool was developed by researchers after reviewing the literature [9, 28–30]. To determine the key challenges of interprofessional collaboration and to explore the best practices for improving interprofessional collaboration. The survey was developed 3 months before use in the current study. To establish face validity and effectively capture the topic under investigation, a survey was submitted to five experts in the critical care nursing department. Each item was assessed using a three-point scale (not necessary, useful but not essential, and essential). The content validity ratio (CVR) is then calculated for each item by employing Lawshe (1975) ‘s method. Items that were not significant at the critical level were eliminated (unclear, double-barreled, and confusing statements were omitted). The survey was piloted on 34 nurses who worked in the critical care unit rather than the selected units involved in the current study. Then the nurses’ responses were entered into a spreadsheet and submitted to the statistics to assess underlying components using principal components analysis (PCA) (± 0.78) and the internal consistency of the survey (Cronbach’s alpha coefficient 0.94). The analysis revealed that the somewhat two items were irrelevant and were dropped. The final form of the survey consists of three parts. Part, one aimed to collect the demographic and clinical data of the nurses who agreed to participate in the study including age, gender, working conditions, years of experience, number of working hours per week, and nurse-to-patient ratio. Part two is concerned with the key challenges that hinder interprofessional collaboration. It consists of 16 statements. The nurses’ responses were collected on a four-point scale of strongly agree [4] and strongly disagree [1], the total scores ranged from 16–64, with high values indicating more challenge-hindered collaborative relationships. Part three consists of two open-ended questions. In this part, the nurses were asked to answer two open-ended questions. The first question was about any other barriers not mentioned in the survey or if they had any comment regarding them (What are other challenges not mentioned in the survey and your feelings toward the professional relation with the physicians?). The second question was about the nurses’ suggestions for improving interprofessional collaboration “What best practices do the nurses suggest for improving interprofessional collaboration in critical care units?”.

### Data collection

- A study has adhered to the Declaration of Helsinki, and this was confirmed and approved by the **Ethical Research Committee at the Faculty of Nursing and the Institutional Review Board of Alexandria University. Reference number**: IRB00013620.
- Permission to conduct the study was obtained from the medical and nursing principles of the selected units after explaining the study’s aims.
- Before data collection, a written informed consent form including research-related information (details of the nature and aims of the research; the expected duration of the nurse’s participation, probable risks and benefits) was submitted to all proposed nurses. Informed consent to voluntary participation was obtained from all the participants in the study.
- Confidentiality was assured by many methods: first by avoiding the collection of any personal data of the participants such as names, and emails which compromised anonymity, for example, names, phone numbers, and email addresses. Second, keep the surveys in a locker, access to the surveys is only allowed for authorized and granted persons (the researchers and the biostatisticians).
- The tool was developed by the researchers and validated by five experts in the field of critical care nursing and piloted for its visibility and clarity.
- The survey was handed to the nurses and collected by the researchers. Nurses were filling out the survey during their break time in the nurse’s break room and in the presence of the researchers.
- Each nurses’ group consisted of between 35 and 40 nurses and each meeting/ interview lasted between 90 and 120 min. Each statement in the survey was explained by the researchers and each nurse’s question was answered before completing the survey or answering the open-ended questions.
- A content analysis was used to identify and extract challenges faced by the nurses in their interprofessional collaboration in the critical care units. It focuses on the interpretation of the similarities and differences within different sections of the text. The nurses’ written texts were prepared by the researchers and were provided to the biostatistician specialized in qualitative data. The basic unit of text was classified, categories and a coding scheme were developed, and all texts were coded. The consistency of the coding was assessed, and findings were extracted. Trustworthiness of the data such as credibility, transferability, dependability, and confirmability were followed.

### Data analysis

Version 23.0 of the IBM SPSS software package was used to feed and analyze data. For comparisons between more than two categories, a one-way ANOVA test was employed. For normally distributed quantitative variables, two categories were compared using the student t-test. To correlate normally distributed quantitative variables, the Pearson coefficient was employed. Linear Regression Analysis Showing the Effect of Demographic on Key Challenges of Interprofessional Collaboration perceived by the nurses participated. The significance of the obtained results was judged at the 5% level.

The second part of the data containing the nurses’ experiences and suggestions for improving the IPC (nurses’ words) was analyzed by the content analysis approach through the following steps: An extensive review of the written text was done several times, line by line and word by word. The text was condensed or shortened while still preserving the core meaning. A condensed text was coded or labeled; a name that most exactly describes the meaning of the text, it was one or two words long. Codes that related to each other were grouped forming categories through their content or context. Finally, the categories were abstracted at an interpretive level to reach the main categories and subcategories.

## Results: part one: quantitative data

### Demographic and clinical data of the participated nurses

Table 1 outlines a comprehensive overview of gender, age, marital status, years of experience, and working hours per week. Among 125 critical care nurses, 89 (71.2%) possess a diploma, while 36 (28.8%) have a bachelor’s degree. Within the diploma holder group, approximately two-thirds were married females, with the remaining one-third being males. The age bracket of 26–30 years represented the largest category in both groups. Additionally, most individuals from both groups were employed in private hospitals and worked over 45 h weekly. Regarding experience, a significant proportion of the diploma nurses had 11 years or more, in contrast to the bachelor nurses who had between 1 and 5 years of experience.

**Table 1:** Demographic & clinical data of the nurses (*n* = 125)

Demographic and Clinical Variables	Diploma (*n* = 89)		Bachelor (*n* = 36)	
	*N*	%	*N*	%
**Age (years)**				
20–25	18	20.2	10	27.8
26–30	35	39.3	13	36.1
31–35	18	20.2	8	22.2
36–40	10	11.2	5	13.9
> 40	8	9.1	0	0
**Gender**				
Female	66	74.2	28	77.8
Male	23	25.8	8	22.2
**Marital Status**				
Single	32	36	22	61.1
Married	57	64	14	38.9
Widow	0		0	0
**Working in a Private Hospital**				
No	21	23.6	8	22.2
Yes	68	70.4	28	77.8
**Years of Experience**				
1–5 Year	18	20.2	11	30.6
6–10-Year	32	36	22	61.1
11 years and above	39	43.8	3	8.3
**Working Hours/Week**				
< 45 h.	27	30.3	10	27.8
> 45 h.	62	69.7	26	72.2

### Key challenges to professional collaboration as perceived by the nurses

Tables 2 and 3 depict the differences in mean values of challenges to professional collaboration as perceived by the nurses. Among the 16 challenges mentioned in the survey, the 𝑡-test analysis and Chi-square (χ^2^) revealed some items agreed by all nurses but with no significant differences and some items agreed by nurses with significant differences in the perceptions toward collaboration between nurses who held bachelor’s degrees and nurses who held diploma degrees. The items that have agreed by the nurses with no significant differences between either category of the nurses are item number 1 (There’s a history of personal conflicts between nurses and physicians) (𝑡-test:1.525; 𝑝 0.133; χ^2^ : 7.147), item number 2 (Physicians don’t trust the competencies of nurses. They think nurses did not receive a good education to decide or share the treatment decisions.) (𝑡-test: 0.546; 𝑝 0.586; χ^2^ : 5.079), item number 3 (Nurses and physicians don’t have shared goals) (𝑡-test:0.0.503; 𝑝 0.616; χ^2^ : 6.313), item number 6 (Physicians and nurses do not have sufficient time for communication and collaboration due to a high workload) (𝑡-test: 0.870; 𝑝 0.386; χ^2^ : 3.860), item number 7 (The hospital administration is more supportive and empowered to physicians than nurses) (𝑡-test:0.0.634; 𝑝 0.527), item number 8 (Nurses do not receive adequate support and appreciation) (𝑡-test:0.0.696; 𝑝 0.488; χ^2^ : 7.495) item number 14 (Physicians do not understand the professional role of nurses) (𝑡-test:0.1.421; 𝑝 0.158; χ^2^ : 2.020).

Moreover, the items that have been agreed by the nurses with no significant differences between either category of the nurses are item number 4 (There’s no interest in collaboration between nurses and physicians. Physicians and nurses do their tasks separately) (𝑡-test:0.3.443; 𝑝 0.001; χ^2^ : 15.111), item number 5 (Physicians are at the top of this hierarchy, so they don’t pay enough attention to nurses’ comments, even if they are right) (𝑡-test:0.11.792; 𝑝 <0.001; χ^2^ : 77.894), item number 9 (Nurses and physicians receive different theoretical and practical backgrounds) (𝑡-test:0.10.361; 𝑝 <0.001; χ^2^ : 62.920), item number 10 (Physicians have the power of authority over nurses (superior-subordinate relationships) (𝑡-test:0.8.869; 𝑝 <0.001; χ^2^ : 53.036), item number 11 (The decision of the treatment plan is only dominated by physicians making it hard for nurses to question the orders even when they are sure these orders have potential detrimental effects on the patient) (𝑡-test:0.8.340; 𝑝 <0.001; χ^2^ : 53.379), item number 12 (Physicians consider nurses their assistants and not their colleagues) (𝑡-test:0.5.290; 𝑝 <0.001; χ^2^ : 36.644), item number 13 (Physicians and nurses have different professional interests and have different perspectives) (𝑡-test:0.5.378; 𝑝 <0.001; χ^2^ : 27.791), item number 15 (Some physicians deal with nurses according to their levels of stress and mood) (𝑡-test:0.2.446; 𝑝 0.018; χ^2^ : 8.675), item number 16 (The primary function of a nurse is to carry out the d physician’s instructions) (𝑡-test:0.10.263; 𝑝 <0.001; χ^2^ : 67.895). Additionally, the total scores and the mean percent score of diploma and bachelor nurses were (Mean:47.80, SD: 3.29 & Mean:38.42, SD: 2.60) & (Mean:39.75, SD: 4.12 & Mean:28.02, SD:3.25). the overall 𝑡-test analysis was (𝑡-test:0.16.856; 𝑝 <0.00).

**Table 2:** Key challenges of interprofessional collaboration perceived by the nurses participated in the study

#	Key Challenges	Diploma (*n* = 89)			Bachelor (*n* = 36)			t	*p*
		Mean	±SD	Rank	Mean	±SD	Rank		
1	There’s a history of personal conflicts between nurses and physicians.	2.91	0.83	**11**	2.61	1.05	**6**	1.525	0.133
2	Physicians don’t trust the competencies of nurses. They think nurses did not receive a good education to decide or share the treatment decisions.	2.60	0.84	**14**	2.50	1.00	**8**	0.546	0.586
3	Nurses and physicians don’t have shared goals.	2.57	1.02	**15**	2.47	1.00	**9**	0.503	0.616
4	There’s no interest in collaboration between nurses and physicians. Physicians and nurses do their tasks separately.	2.20	0.88	**16**	1.61	0.84	**15**	3.443*	0.001*
5	Physicians are at the top of this hierarchy, so they don’t pay enough attention to nurses’ comments, even if they are right.	2.85	0.75	**13**	1.28	0.45	**16**	11.792*	< 0.001*
6	Physicians and nurses do not have sufficient time for communication and collaboration due to a high workload.	3.00	0.80	**9**	3.14	0.83	**3**	0.870	0.386
7	The hospital administration is more supportive and empowered to physicians than nurses.	3.06	0.86	**8**	3.17	0.94	**2**	0.634*	0.527*
8	Nurses do not receive adequate support and appreciation.	2.98	0.78	**10**	2.86	0.99	**4**	0.696	0.488
9	Nurses and physicians receive different theoretical and practical backgrounds.	3.21	0.63	**4**	1.83	0.77	**14**	10.361*	< 0.001*
10	Physicians have the power of authority over nurses (superior-subordinate relationships).	3.18	0.56	**5**	2.14	0.68	**10**	8.869*	< 0.001*
11	The decision of the treatment plan is only dominated by physicians making it hard for nurses to question the orders even when they are sure these orders have potential detrimental effects on the patient.	3.08	0.69	**7**	1.92	0.73	**12**	8.340*	< 0.001*
12	Physicians consider nurses their assistants and not their colleagues.	3.42	0.56	**1**	2.58	0.87	**7**	5.290*	< 0.001*
13	Physicians and nurses have different professional interests and have different perspectives.	2.90	0.75	**12**	2.11	0.71	**11**	5.378*	< 0.001*
14	Physicians do not understand the professional role of nurses.	3.42	0.50	**1**	3.56	0.50	**1**	1.421	0.158
15	Some physicians deal with nurses according to their levels of stress and mood.	3.12	0.69	**6**	2.72	0.88	**5**	2.446*	0.018*
16	The primary function of a nurse is to carry out the d physician’s instructions.	3.30	0.65	**3**	1.92	0.77	**12**	10.263*	< 0.001*
**Total score**		**47.80**	**3.29**		**38.42**	**2.60**		16.856*	< 0.001*
**Mean Percent score**		**39.75**	**4.12**		**28.02**	**3.25**			

t: Student t-test. *: Statistically significant at *p* ≤ 0.05

**Table 3:** Part two: key challenges of interprofessional collaboration perceived by the nurses participated in the study

		Diploma (*n* = 89)								Bachelor (*n* = 36)								χ^2^	^MC^ *p*
#	Key Challenges	Strongly Disagree		Disagree		Agree		Strongly Agree		Strongly Disagree		Disagree		Agree		Strongly Agree			
		No.	%	No.	%	No.	%	No.	%	No.	%	No.	%	No.	%	No.	%		
1	There’s a history of personal conflicts between nurses and physicians.	4	4.5%	23	25.8%	39	43.8%	23	25.8%	7	19.4%	8	22.2%	13	36.1%	8	22.2%	7.147	0.067
2	Physicians don’t trust the competencies of nurses. They think nurses are not educated enough to decide or share treatment decisions.	10	11.2%	26	29.2%	43	48.3%	10	11.2%	6	16.7%	13	36.1%	10	27.8%	7	19.4%	5.079	0.158
3	Nurses and physicians don’t have shared goals.	16	18.0%	25	28.1%	29	32.6%	19	21.3%	9	25.0%	5	13.9%	18	50.0%	4	11.1%	6.313	0.097
4	There’s no interest in collaboration between nurses and physicians. Physicians and nurses do their tasks separately.	20	22.5%	38	42.7%	24	27.0%	7	7.9%	21	58.3%	9	25.0%	5	13.9%	1	2.8%	15.111*	0.002*
5	Physicians are at the top of this hierarchy, so they don’t pay enough attention to nurses’ comments, even if they are right.	3	3.4%	23	25.8%	47	52.8%	16	18.0%	26	72.2%	10	27.8%	0	0.0%	0	0.0%	77.894*	< 0.001*
6	Physicians and nurses do not have sufficient time for communication and collaboration due to a high workload.	2	2.2%	22	24.7%	39	43.8%	26	29.2%	2	5.6%	4	11.1%	17	47.2%	13	36.1%	3.860	0.262
7	The hospital administration is more supportive and empowered to physicians than nurses.	2	2.2%	24	27.0%	30	33.7%	33	37.1%	4	11.1%	1	2.8%	16	44.4%	15	41.7%	13.819*	0.002*
8	Nurses do not receive adequate support and appreciation.	4	4.5%	16	18.0%	47	52.8%	22	24.7%	6	16.7%	2	5.6%	19	52.8%	9	25.0%	7.495	0.058
9	Nurses and physicians receive different theoretical and practical backgrounds.	0	0.0%	10	11.2%	50	56.2%	29	32.6%	14	38.9%	14	38.9%	8	22.2%	0	0.0%	62.920*	< 0.001*
10	Physicians have the power of authority over nurses (superior-subordinate relationships).	0	0.0%	7	7.9%	59	66.3%	23	25.8%	6	16.7%	19	52.8%	11	30.6%	0	0.0%	53.036*	< 0.001*
11	The decision of the treatment plan is only dominated by physicians making it hard for nurses to question the orders even when they are sure these orders have potential detrimental effects on the patient.	2	2.2%	12	13.5%	52	58.4%	23	25.8%	10	27.8%	20	55.6%	5	13.9%	1	2.8%	53.379*	< 0.001*
12	Physicians consider nurses their assistants and not their colleagues.	0	0.0%	3	3.4%	46	51.7%	40	44.9%	3	8.3%	15	41.7%	12	33.3%	6	16.7%	36.644*	< 0.001*
13	Physicians and nurses have different professional interests and have different perspectives.	4	4.5%	18	20.2%	50	56.2%	17	19.1%	6	16.7%	21	58.3%	8	22.2%	1	2.8%	27.791*	< 0.001*
14	Physicians do not understand the professional role of nurses.	0	0.0%	0	0.0%	52	58.4%	37	41.6%	0	0.0%	0	0.0%	44.4%	20	55.6%	16	2.020	0.155
15	Some physicians deal with nurses according to their levels of stress and mood.	1	1.1%	13	14.6%	49	55.1%	26	29.2%	3	8.3%	11	30.6%	15	41.7%	7	19.4%	8.675*	0.029*
16	The primary function of a nurse is to carry out the d physician’s instructions.	1	1.1%	6	6.7%	47	52.8%	35	39.3%	11	30.6%	18	50.0%	6	16.7%	1	2.8%	67.895*	< 0.001*

χ^2^: Chi-square test MC: Monte Carlo *: Statistically significant at *p* ≤ 0.05

**Fig. 1:**
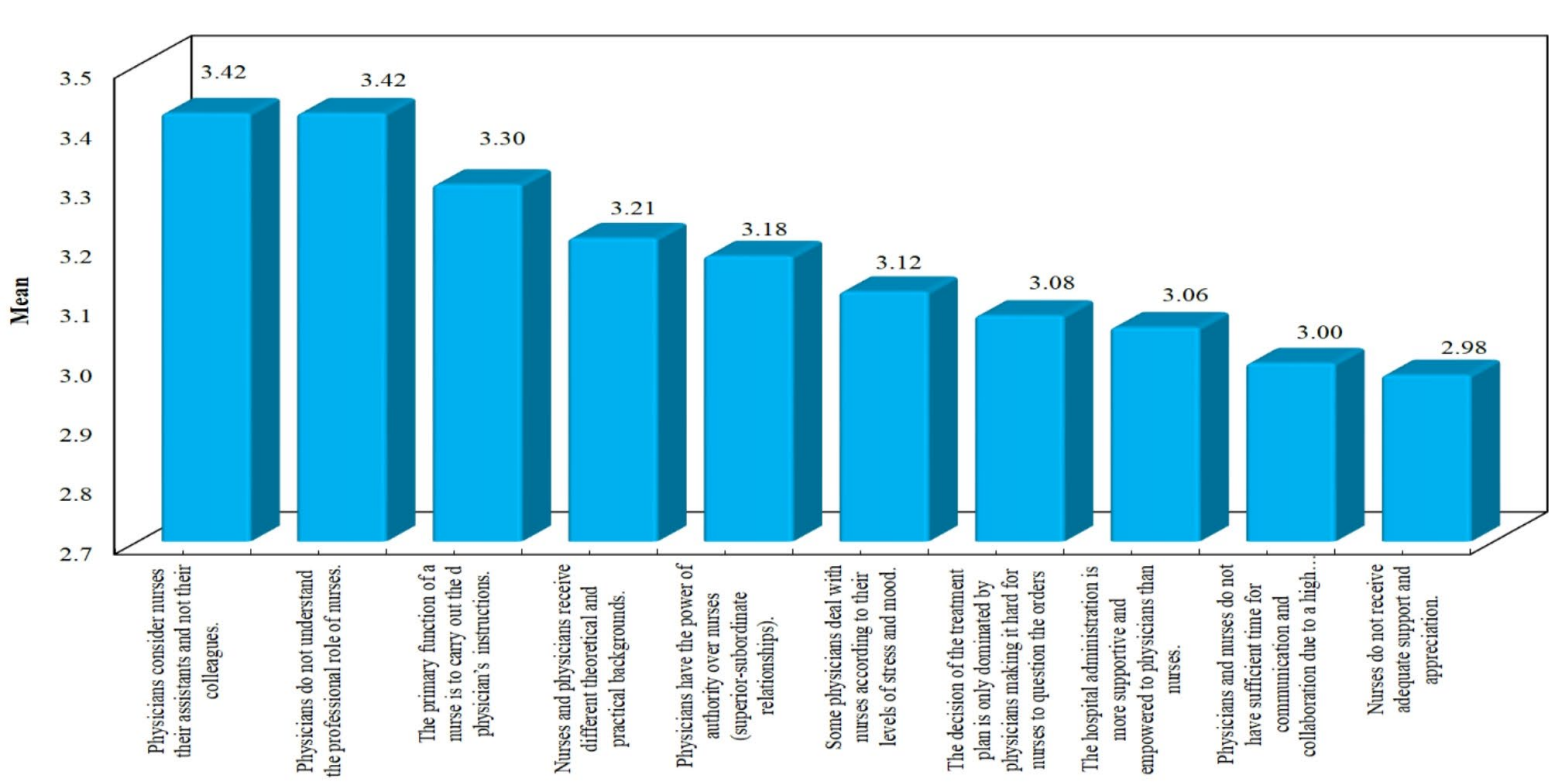
The top ten challenges to interprofessional collaboration perceived by nurses with a diploma degree

**Fig. 2:**
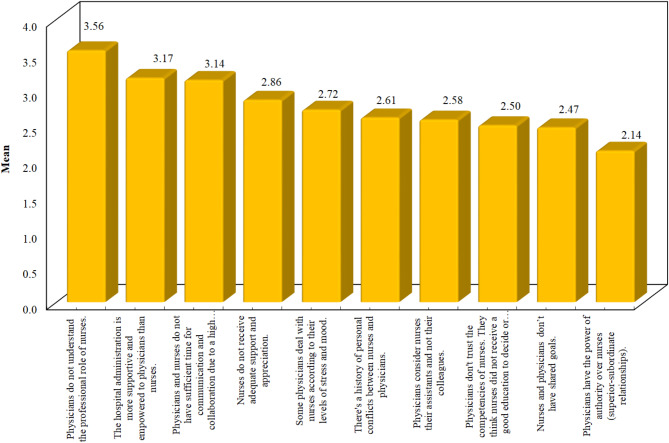
The top ten challenges to interprofessional collaboration perceived by nurses with a bachelor’s degree

Figures 1 and 2 **Illustrate the ranking of the key challenges perceived by the nurses according to their level of education.** Analysis of the figures showed that item number 14 (Physicians do not understand the professional role of nurses) had been ranked as the highest challenge hindering interprofessional collaboration by the majority of the nurses with diplomas and bachelor’s degrees (Mean:3.42, SD:0.50) & (Mean = 3.56, SD = 0.50) respectively. Among the top ten items, the nurses with diploma degrees perceived “Nurses do not receive adequate support and appreciation” as number ten among the other challenges hindering the IPC (Mean:2.98, SD:0.78), while the nurses with bachelor’s degrees perceived “Physicians have the power of authority over nurses (superior-subordinate relationships” as number ten among the other challenges hindering the IPC (Mean:3.18, SD:0.56).

Table 4 **shows the stepwise hierarchical linear regression analysis of the correlation between the demographic and clinical data of the nurses and the key challenges to ICP.** Significant correlations have been detected between the level of nurses’ education, limited time as a result of workload, and the perceptions of the key challenges hindering the IPC.

**Table 4:** Correlation between demographic and clinical data of the nurses and the key challenges to interprofessional collaboration

Demographic/Clinical Variables	B	Beta	t	*p*	95% CI	
					LL	UL
**Step 1**						
**Level of Education**	-9.381	-0.809	-15.262*	< 0.001*	-10.598	-8.164
**R**^**2**^ **= 0.654**,** adjusted R**^**2**^ **= 0.652**, F = 232.939^*^, *p* > 0.001^*^						
**Step 2**						
**Level of Education**	-9.247	-0.797	-15.213*	< 0.001*	-10.451	-8.044
**Working Hours/Week**	-1.458	-0.117	-2.235*	0.027*	-2.749	-0.167
**R**^**2**^ **= 0.668**,** adjusted R**^**2**^ **= 0.663**, F = 122.753^*^, *p* > 0.001^*^						

**F**,** p: f and p** values for the model **R**^**2**^: Coefficient of determination **B**: Unstandardized Coefficients **Beta**: Standardized Coefficients

.

### Part two: analysis of the open-ended questions

As regards the analysis of the open-ended questions, and in response to the question of other challenges and feelings toward the IPC, “the words inferiority, or discrimination and inequity were repeated by the majority of the diploma nurses in their written texts”. The following are some quotes extracted from nurses’ texts. One nurse mentioned “Yes, it is a long history of unsupported and unappreciated nurses’ performance” “Nurse is not always supported or appreciated”, “We are considered as servants for the physicians”, “Physicians see nurses as “extras” or “assistants, followers”, “Some physicians see us didn’t have enough scientific knowledge to share with them the treatment plan of the patients, it is better”, “When a problem happened to my patient, I always the person who received the blame, and physician is considered as an angel”. However, some nurses objected and refused these feelings, and they mentioned, “No one can make you feel inferior without your consent”. The feeling of conflict was raised among Baccalaureate nurses who refused the feeling of inferiority. They found themselves graduating from faculties, holding bachelor’s degrees same as the physicians. They mentioned they deserve respect, appreciation, and support from the physicians, hospital administration, and the community.

Table 5 highlights key categories and recommendations that nurses believe are essential for enhancing interprofessional collaboration in critical care units. The majority of nurses identified five main categories as best practices, which have been prioritized based on their viewpoints. Notably, the category “Changing physician attitudes toward nurses” is viewed by the nurses as the top suggestion for best practices and includes four implementation recommendations. Furthermore, “Role clarification” is regarded as the second most important suggestion for advancing interprofessional collaboration, consisting of two recommendations for implementation. Additionally, nurses recognize “Improving communication skills” as an important suggestion for enhancing interprofessional collaboration among the critical care team, comprising three implementation recommendations. The category “Interprofessional conflict resolution,” which includes four suggested implementations, is seen by nurses as the fourth category for improving collaboration. Lastly, nurses view support and motivation as another suggestion that, if adhered to, will foster professional collaboration in critical care units.

**Table 5:** Nurses’ suggestions for best practices for nurses’-physicians collaboration

Code #	Categories	Suggestions for Implementation
1.	Changing physician attitudes toward nurses	− View a nurse as a partner and peer of a physician, rather than just an assistant. − Foster a culture that encourages collaboration, mutual respect, confidence, and collective objectives among nurses and physicians. − Revise the working relationship between nurses and physicians from a superior-subordinate dynamic to a collaborative one. − Show respect for professional boundaries while maintaining appropriate relationships between nurses and physicians. − Make sure to involve physicians and nurses in the decision-making process and utilize their expertise to attain positive patient healthcare outcomes.
2.	Role clarification	− Conduct an orientation session for recently employed nurses and physicians to raise the awareness of the physicians and nurses regarding the scope of practice of each other. − Respect, understand, and value the nurses ‘roles, responsibilities, and competencies. Physicians could consult the nurses, involve them, and respect their opinions, and suggestions they contributed during patient care discussions or rounds.
3.	Improving communication skills	− Adopt effective formal communication tools to enable conversations and interactions that improve team performance. − Tackle the problems of physician and nurse shortages and heavy workloads that inhibit effective communication and collaboration. − Advise physicians to speak loudly and clearly so that nurses can hear and follow the discussion during the medical rounds.
4.	Interprofessional Conflict Resolution	− Think about the healthcare team’s implicit biases and their approach to managing conflicting viewpoints and create and apply strategies to deal with conflict. − Recognize the common situations that may lead to disagreements or conflicts, involve role ambiguity, power imbalances, and varying goals, and set constructive steps to prevent them. − Establish a safe environment in which to express diverse opinions. − Establish a level of consensus among interprofessional teams with different perspectives, ensuring that all of them feel that their opinions have been acknowledged to enhance patient care and outcomes.
5.	Support and Motivation	− Boost nurses’ morale by cultivating positive working connections, equipping the staff with knowledge, and introducing strategies to mitigate stress. − Encourage and support nurses to pursue further studies and participate in in-service training focused on current critical care issues and protocols, as well as joining journal clubs to enhance their knowledge. Nurses have proposed that gaining adequate knowledge can lead to increased confidence, assertiveness, and autonomy. − Support nurses’ roles by granting administrative authority to empower their autonomy and authority.

## Discussion

Nurses and physicians in critical care teams work closely together, supporting and enhancing each other’s efforts. They share common objectives and are dedicated to fostering a secure and supportive working environment for high-quality nursing care. Yet, a lack of understanding of their roles has led to frequent conflicts between nurses and physicians in critical care settings. Resolving these challenges is essential to safeguarding the safety and quality of care for critically ill patients. For this reason, the current study aims to determine the key challenges that hinder interprofessional collaboration and explore the best practices to eliminate or solve these challenges for moving forward in the nurses’ and physicians’ interprofessional relationship as they are perceived by the nurses. To fulfill these aims, a group of nurses was recruited to participate and share their perceptions toward their collaboration with the physicians and suggest solutions to eliminate the challenges that hinder effective collaboration.

### Analysis of the quantitative part of the survey

An unexpected finding was the varying views of the participating nurses on the concept of interprofessional collaboration based on their educational background. The analysis of the challenges survey revealed significant differences between nurses with baccalaureate degrees and those with diploma degrees. Particularly, there were discrepancies in the statements highlighting nurses as subservient to physicians and their roles being seen as followers who carry out physicians’ orders without negotiation. Nurses with baccalaureate degrees strongly opposed being subordinate to physicians, asserting that they are equally educated and qualified to carry out their responsibilities. They also referenced historical nurse-physician interactions, noting the historical dominance of physicians and the subsequent subservience of nurses in these interactions.

Otherwise, nurses holding diploma degrees generally did not face challenges in collaborating with physicians, indicating their acceptance of the collaborative relationship. Most of them acknowledged their main role as carrying out physicians’ instructions. They attributed this acknowledgment to their educational preparation to unquestionably follow physicians’ orders and the institutional leadership’s emphasis on physicians’ sole responsibility for patient treatment decisions, making it difficult for nurses to question orders even when they perceive potential harm to the patient. Additionally, nurses expressed their reluctance to challenge physicians’ authority, though they found justification for questioning some of the physicians’ decisions in advocating for patients (superior-subordinate relationships).

The findings of the current study are consistent with a study by Aghamohammadi et al. (2019). They conducted the study in Iran and showed that the dominance of physicians and considering nurses as their assistants and not their colleagues could negatively influence nurse-physician collaboration [7]. They advised nurses to assume additional responsibilities in the decision-making aspect of patient care to promote successful inter-professional collaboration. In a study by Aymen and colleagues (2017), it was highlighted that historically, doctors have considered nurses to be subordinates and recipients of orders for execution. This traditional viewpoint suggests that the relationship between physicians and nurses could impact the attitudes of healthcare professionals toward collaboration. Nevertheless, nurses holding diploma degrees are regarded as assistants to physicians, with their primary responsibility being to aid physicians and adhere to this division of duties. This disparity may have stemmed from differing perceptions of their roles in clinical environments, cultural variations, and individual attributes [31].

A similar pattern of results was obtained in a study conducted in Ireland, the USA and Ethiopia showed that physicians are more likely to believe in a traditional “captain of the ship” relationship and tend to consider nurses as inferior rather than as colleagues. Furthermore, nurses believe that they tend to be unwilling to challenge physicians’ authority. Along the same line, Dahlawi et al. (2023) found that around 31% of participants perceive a gap in physicians’ understanding of nurses’ roles and responsibilities as a nurse and cite ongoing problems with communication and collaboration [21, 26, 32].

Moreover, the majority of nurses (diploma & bachelor) agreed and strongly agreed without significant difference regarding the presence of a history of personal conflicts between nurses and physicians, physicians don’t trust the competencies of nurses. They think nurses are not educated enough to decide or share treatment decisions, Nurses and physicians don’t have shared goals, physicians and nurses do not have sufficient time for communication and collaboration due to a high workload, nurses do not receive adequate support and appreciation, and physicians do not understand the professional role of nurses. Furthermore, a lack of understanding of the competencies of other professions, resistance toward change, and the lack of interprofessional education in educational programs have all been noted as barriers to interprofessional collaboration practice reported by Josi [33].

In 2015, Choe and colleagues discovered that nurses were stressed because physicians didn’t include them in important tasks such as care planning, decision-making, and communicating with patients and their families. On the other hand, nurses felt frustrated and unappreciated because they were not involved in communication and decision-making, which affected the relationship between nurses and physicians and the nurse’s ability to provide patient-centered care [2].

To determine if there is a significant correlation between the variables ((demographic and clinical data of the nurses and the challenges to ICP), A stepwise hierarchical linear regression analysis shows a significant correlation existed between the nurses’ perceived challenges to IPC and the level of education, and heavy workload that limit the time for communication and collaboration. Nurses agreed that insufficient time due to a high workload is considered a hindrance to collaboration with the physician. The heavy workload could be explained by the nurses’ shortage. As a result of economic burdens, nurses may travel abroad to work in other countries.

The correlation findings from the present study align with those of numerous other studies. For instance, Tang et al. (2018) discovered that demanding workloads presented a challenge due to high clinical load and restricted professional communication and collaboration [34]. Similarly, In Lebanon, a study was carried out to compare the views of nurses and doctors on their collaborative work experiences. According to the study findings, time constraints emerged as the most commonly experienced and top-ranked obstacle [21]. In a Norwegian hospital, a qualitative study was conducted to investigate the collaboration between nurses and doctors in monitoring and treating patients in the surgical ward. The findings indicated that there were limited opportunities for interprofessional collaboration due to staff shortages and restricted collaboration time [35].

Contrary to this result, the study conducted by [21] Found that more than 75% of physicians believed that nurses could be involved in making policy decisions affecting their working conditions. While they perceive that the physician’s role is the most important one in the health team. This reflects the physician’s respect for the nurse’s role due to carrying out his orders without arguments and interference from her which showed the physician’s superiority over nurses. However, around 31% of participants perceive a gap in physicians’ understanding of nurses’ roles and responsibilities as a nurse and cite ongoing problems with communication and collaboration [26].

### Analysis of the open-ended questions

As regards the analysis of the open-ended questions, and in response to the question of other challenges and feelings toward the IPC, “the words inferiority and inequity were repeated by the majority of the nurses in their written texts”. The feeling of inferiority that nurses felt could be attributed to many reasons. The historical textual and epigraphical evidence highlights the progressive role of physicians and the assistance provided by nurses in ancient Egypt. Nurses were perceived as followers of physicians and were given a minor professional position due to gender-related role differences. Physicians held the top position in the hierarchy and expected nurses to strictly follow their orders without discussions or negotiations. They often disregarded nurses’ input, even when it was valid, due to a lack of understanding of their educational backgrounds and roles, particularly those with baccalaureate degrees. Furthermore, the hospital administration was more supportive of and empowered physicians over nurses. Additionally, nurses did not receive sufficient support and acknowledgment from the administration, as evidenced by the fact that they were solely held accountable and punished for medical errors [4, 5, 30, 31, 36, 37].

Furthermore, the nurses’ feeling of inequity with physicians, especially in the disciplinary actions, could be explained by the following facts. Complaints made by nurses against physicians are disregarded by the administration, with blame being privately assigned to physicians for any mistakes, while the opposite is true for nurses. Additionally, nurses are unable to voice their objections to physicians’ violations of policies and regulations and often become the victims of physicians’ errors or neglect. These circumstances all serve to demonstrate the authority physicians hold over nurses and their efforts to maintain a sense of superiority. This conclusion is supported by findings from other studies, which have established that critical care nurses are underappreciated, physicians do not consistently value their opinions or patient assessments, and the nurses may feel marginalized during decision-making discussions [38–40]. In addition, an analysis of the nurses’ responses concerning the second open-ended question about the best practices for moving forward revealed that 5 categories were identified, these include physician attitudes toward the nurses, communication tools and skills, nurses’ roles clarification, interprofessional conflict resolution, and support and motivation.

### Limitations

The sample included nurses only, future studies could benefit from including physicians to determine the physicians’ perspectives. Additionally, to improve representativeness and generalizability, future research should include a bigger sample.

## Conclusion

The current study highlights several key findings:

- On one hand, unlike diploma nurses, nurses with baccalaureate degrees reported disagreement with the level of collaboration between nurses and physicians, especially in the statements of the physicians’ dominance of the decision, their position at the top of the hierarchy, consideration of the nurses as followers not as colleagues, and the power of authority that the physicians have over nurses (superior-subordinate relationships).
- On the other hand, feelings of inferiority and inequality were reported by the nurses as a response to the open-ended question.
- In addition, 5 categories have been suggested by the nurses as best practices for moving forward in their collaboration with the physicians, these include physician attitudes toward the nurses, communication tools and skills, nurses’ roles clarification, interprofessional conflict resolution, and support and motivation.
- The results of this study will assist in determining the challenges and exploring the strategies that can strengthen the IPC between physicians and nurses. Overcoming the perceived challenges and implementing the strategies to improve the IPC will enhance and promote patient safety by reducing medical errors, improving the quality of care, and patient outcomes, and enhancing workplace conditions, cost efficiency, and the satisfaction levels of healthcare professionals.

### Recommendations

Based on the results of this study, the researchers would like to suggest the following recommendations:

### Recommendations for nursing practice/ management

- Nurture a culture of teamwork and cooperation among nurses and physicians as well as with other allied health care providers in the critical care units.
- Improve teamwork and cooperation by fostering effective communication and interpersonal skills among nurses, physicians, and other allied healthcare providers.
- Show respect for one another and recognize the scope of practice for each discipline, as well as the distinctions in roles. Treat each other as colleagues to promote an inclusive and collaborative work environment.
- Equip nurses with the latest knowledge and clinical skills related to critical care management, professional communication abilities, and a collaborative attitude towards working with physicians by participating in conferences, seminars, workshops, reading journals, and engaging in in-service education.
- Support, motivate, and appreciate the nurses’ performance to improve their professional dignity and satisfaction.
- Held seminars to physicians about the deteriorated effect of practicing dominant authority on staff nurses; many nurses tend to leave the profession because of such practices.
- Empower nursing staff with adequate authority.
- Conduct a well-planned orientation program for physicians and nurses, which covers the roles of the health team members, policies, and procedures.
- Tackle the common sources of the conflict raised between the nurses and the physicians and adopt appropriate conflict resolution.
- Apply fair disciplinary action equally to nurses and physicians.

### Recommendations for nursing education

- Enhance the concepts of collaboration teamwork, and communication in the nursing and medical curriculums.
- Equip the student nurses with knowledge and clinical skills, and a positive attitude toward collaboration with the physicians and other healthcare members.
- Involve medical and nursing students in clinical scenarios about teamwork and collaboration allowing them to understand their respective roles and develop trust between the physicians and nurses.

### Recommendations for nursing research

- Further research is needed to determine and compare nurses’ and physicians’ perceptions and opinions regarding their collaboration in the critical care units.
- Interventional studies are required to bridge the gap between nurses’ and physicians’ collaborative relationships in Egypt.

## Electronic supplementary material

Below is the link to the electronic supplementary material.

Supplementary Material 1

## Data Availability

The datasets analyzed during the current study are available from the corresponding authors upon reasonable request.
