# Supplementary material for: Challenges and best practices for moving forward in interprofessional collaboration in critical care units: nurses’ perspectives
Source: BMC Nurs. 2025 Mar 25;24:317. doi: 10.1186/s12912-025-02860-0 (PMC11934773; doi:10.1186/s12912-025-02860-0)
Supplement: Supplementary file 1 — Supplementary Material 1 [file 12912_2025_2860_MOESM1_ESM.pdf]

Critical Care Nurse' Perspectives Toward Interprofessional Collaboration: Key Challenges and Best Practices for Moving Forward

Dear colleague:

You are invited to participate in a research study entitled "Critical Care Nurse' Perspectives Toward Interprofessional Collaboration: Key Challenges and Best Practices for Moving Forward" This research was conducted by Dr. Amina Hamida Salem, Assistant Professor, Assistant Professor, Department of Critical Care and Emergency Nursing, and Professor Dr. Hala Ahmed Abdo, Professor, Department of Nursing Administration, Faculty of Nursing, Alexandria University. You have been selected as a potential participant in this research because you work directly with physicians in critical care units and are in an ideal position to provide us with valuable direct information about the environment of your professional relationship with the physicians.

If you decide to participate in this research study by completing the attached questionnaire, please note that there are no risks or inconveniences expected and there are no financial incentives. However, your participation will be a valuable addition to my study and the results could lead to dissemination through academic journal articles for application and adoption to maintain patient safety. Knowing that any information obtained will remain confidential and will not be disclosed except with your permission.

Your signature indicates that you have read and understood the information provided above and that you willingly agree to participate. finally, I would like to assure you that any personal data will be confidential.

Name:

Signature:

Part one: Demographic & Clinical Data of the Nurses

1. **Age (Year)**
- ☐ 25- **20**
- ☐ 30 – 26
- ☐ 35 – 31
- ☐ 40 - 36
- ☐ 40>

2. **Marital status**
- ☐ Single
- ☐ Married
- ☐ Widow
- ☐ Divorced

2. **Gender**
- ☐ Male
- ☐ Female

3. **Years of experience**
- ☐ <5 years
- ☐ 5 -10 years
- ☐ 11- 20 years
- ☐ >20 years

4. **Level of Education**
- ☐ Diploma
- ☐ Bachelor
- ☐ Postgraduate

5. **Working in a Private Hospital**
- ☐ Yes
- ☐ No

6. **Working Hours/Week**
- ☐ < 45 hrs.
- ☐ > 45 hrs.

Part two: Key Challenges of Interprofessional Collaboration perceived by the nurses participated in the study

#	Items	Strongly Agree (4)	Agree (3)	Disagree (2)	Strongly Disagree (1)
**1.**	There's a history of personal conflicts between nurses and physicians.				
**2.**	Physicians don't trust the competencies of nurses. They think nurses did not receive a good education to decide or share the treatment decisions.				
**3.**	Nurses and physicians don't have shared goals.				
**4.**	There's no interest in collaboration between nurses and physicians. Physicians and nurses do their tasks separately.				
**5.**	Physicians are at the top of this hierarchy, so they don't pay enough attention to nurses' comments, even if they are right.				
**6.**	Physicians and nurses do not have sufficient time for communication and collaboration due to a high workload				
**7.**	The hospital administration is more supportive and empowered to physicians than nurses.				

#	Items	Strongly Agree (4)	Agree (3)	Disagree (2)	Strongly Disagree (1)
**8.**	Nurses do not receive adequate support and appreciation.				
**9.**	Nurses and physicians receive different theoretical and practical backgrounds.				
**10.**	Physicians have the power of authority over nurses (superior-subordinate relationships).				
**11.**	The decision of the treatment plan is only dominated by physicians making it hard for nurses to question the orders even when they are sure these orders have potential detrimental effects on the patient.				
**12.**	Physicians consider nurses their assistants and not their colleagues.				
**13.**	Physicians and nurses have different professional interests and have different perspectives.				
**14.**	Physicians do not understand the professional role of nurses.				
**15.**	Some physicians deal with nurses according to their levels of stress and mood.				
**16.**	The primary function of a nurse is to carry out the d physician’s instructions.				

Please read and think carefully before providing your answers.

1. **What are other challenges that were not mentioned in the survey and your feelings toward the professional relation with the physicians?**

2. **What best practices do the nurses suggest for improving interprofessional collaboration in critical care units?**

End of the Survey

Thank you for your time
